# Supplementary material for: Sociodemographic profile, functionality, depression, and frailty as determinants for the risk of abuse and violence against older people in the community: An observational study conducted in Brazil
Source: PLoS One. 2025 Jun 16;20(6):e0317855. doi: 10.1371/journal.pone.0317855 (PMC12169517; doi:10.1371/journal.pone.0317855)
Supplement: S1 Table — (DOCX) [file pone.0317855.s001.docx]

**S1 Table. Detailed logistic regression analysis of the most significant aspects and variables, adjusted for potential confounding factors according to age groups.**

| **Risk of abuse and violence (HS-EAST)** | |  | **Younger (n= 132)** | | | | | **Older (n=68)** | | | | | |
| --- | --- | --- | --- | --- | --- | --- | --- | --- | --- | --- | --- | --- | --- |
|  |  |  | **R^2^** ^a^ | **p** ^b^ | **ß (S.E)** | **Wald** | **OR (CI 95%)** | **R^2^** | **p** ^b^ | **ß (S.E)** | **Wald** | **OR (CI 95%)** | |
| **Aspects evaluated** | | | | | | | | | | | | |  |
| **Functionality (Lawton & Brody) - Crude** | Stage 1 | | 0.17 | <0.001 | -0.31 (0.10) | 12.10 | 0.7 (0.6 – 0.9) | 0.35 | <0.001 | -0.30 (0.10) | 14.20 | 0.7 (0.6 – 0.9) | |
|  | Constant | | - | 0.001 | 5.80 (1.76) | 10.82 | 325.3 | - | 0.001 | 4.74 (1.40) | 11.36 | 113.9 | |
| Adjusted by Skin color | No white ^b^ | Stage 1 | 0.17 | 0.023 | -0.35 (0.15) | 5.19 | 0.7 (0.5 – 0.9) | 0.49 | 0.037 | -0.53 (0.26) | 4.37 | 0.6 (0.3 – 1.0) | |
|  |  | Constant | - | 0.023 | 6.82 (2.99) | 5.19 | 914.1 | - | 0.40 | 9.95 (4.84) | 4.22 | 20872.4 | |
|  | White | Stage 1 | 0.16 | 0.010 | 0.28 (0.11) | 6.68 | 0.7 (0.6 – 0.9) | 0.29 | 0.005 | -0.27 (0.09) | 8.04 | 0.8 (0.6 – 0.9) | |
|  |  | Constant | - | 0.021 | 4.90 (2.12) | 5.31 | 133.7 | - | 0.23 | 3.66 (1.60) | 5.20 | 38.8 | |
| Adjusted by Education | Literate ^c^ | Stage 1 | 0.12 | 0.005 | -0.28 (0.10) | 7.73 | 0.8 (0.6 – 0.9) | 0.24 | 0.007 | -0.26 (0.10) | 7.23 | 0.8 (0.6 – 0.9) | |
|  |  | Constant | - | 0.011 | 5.03 (1.98) | 6.44 | 152.9 | - | 0.035 | 3.58 (1.70) | 4.45 | 35.9 | |
|  | Illiterate | Stage 1 | - | - | - | - | - | 0.31 | 0.058 | -0.30 (0.16) | 3.60 | 0.7 (0.5 – 1.0) | |
|  |  | Constant | - | - | - | - | - | - | 0.042 | 5.53 (2.72) | 4.14 | 253.4 | |
| **Depressive Symptoms (GDS-15) - Crude** | Stage 1 | | 0.46 | <0.001 | 0.56 (0.10) | 32.65 | 1.7 (1.4 – 2.1) | 0.33 | <0.001 | 0.43 (0.12) | 13.60 | 1.5 (1.2 – 2.0) | |
|  | Constant | | - | <0.001 | -2.34 (0.42) | 30.47 | 0.01 | - | <0.001 | 2.23 (0.59) | 14.25 | 0.11 | |
| Adjusted by Skin color | No white ^b^ | Stage 1 | 0.43 | <0.001 | 0.51 (0.13) | 14.39 | 1.6 (1.6 – 2.2) | 0.40 | 0.015 | 0.46 (0.19) | 5.97 | 1.6 (1.1 – 2.3) | |
|  |  | Constant | - | 0.001 | -1.96 (0.60) | 10.53 | 0.1 | - | 0.089 | -1.33 (0.79) | 2.88 | 0.3 | |
|  | White | Stage 1 | 0.45 | <0.001 | 0.59 (0.14) | 17.20 | 1.8 (1.3 – 2.4) | 0.32 | 0.007 | 0.47 (0.17) | 7.23 | 1.6 (1.1 – 2.3) | |
|  |  | Constant | - | <0.001 | -2.63 (0.61) | 18.80 | 0.1 | - | 0.001 | -2.98 (0.92) | 10.43 | 0.1 | |
| Adjusted by Education | Literate ^c^ | Stage 1 | 0.42 | <0.001 | 0.54 (0.11) | 25.37 | 1.7 (1.4 – 2.1) | 0.30 | 0.005 | 0.42 (0.15) | 7.80 | 1.5 (1.1 – 2.0) | |
|  |  | Constant | - | <0.001 | -2.40 (0.47) | 26.59 | 0.1 | - | 0.001 | -2.75 (0.79) | 12.00 | 0.1 | |
|  | Illiterate | Stage 1 | 0.54 | 0.027 | 0.69 (0.31) | 4.89 | 2.0 (1.1 – 3.7) | 0.31 | 0.043 | 0.45 (0.22) | 4.09 | 1.6 (1.0 – 2.4) | |
|  |  | Constant | - | 0.082 | -1.95 (1.12) | 3.02 | 0.1 | - | 0.228 | -2.1 (1.0) | 1.45 | 0.3 | |
| **Frailty (EFS) - Crude** | Stage 1 | | 0.19 | <0.001 | 0.27 (0.07) | 16.44 | 1.3 (1.2 – 1.5) | 0.24 | <0.001 | 0.27 (0.08) | 10.67 | 1.3 (1.1 – 1.6) | |
|  | Constant | | - | <0.001 | -1.41 (0.35) | 16.47 | 0.24 | - | 0.001 | -1.98 (0.58) | 11.77 | 0.14 | |
| Adjusted by Skin color | No white ^b^ | Stage 1 | 0.15 | 0.019 | 0.25 (0.11) | 5.46 | 1.3 (1.0 – 1.6) | 0.44 | 0.012 | 0.43 (0.17) | 6.26 | 1.5 (1.1 – 2.1) | |
|  |  | Constant | - | 0.060 | -1.07 (0.57) | 3.55 | 0.3 | - | 0.053 | -1.77 (0.41) | 3.76 | 0.2 | |
|  | White | Stage 1 | 0.20 | 0.002 | 0.27 (0.01) | 9.81 | 1.3 (1.1 – 1.5) | 0.16 | 0.033 | 0.23 (0.11) | 4.54 | 1.3 (1.0 – 1.6) | |
|  |  | Constant | - | <0.001 | -1.60 (0.44) | 12.99 | 0.2 | - | 0.004 | -2.28 (0.80) | 8.10 | 0.1 | |
| Adjusted by Education | Literate ^c^ | Stage 1 | 0.15 | 0.001 | 0.25 (0.07) | 10.97 | 1.3 (1.1 – 1.5) | 0.18 | 0.020 | 0.26 (0.11) | 5.37 | 1.3 (1.0 – 1.6) | |
|  |  | Constant | - | <0.001 | -1.40 (0.37) | 14.68 | 0.25 | - | 0.002 | -2.35 (0.75) | 9.87 | 0.1 | |
|  | Illiterate | Stage 1 | - | - | - | - | - | - | - | - | - | - | |
|  |  | Constant | - | - | - | - | - | - | - | - | - | - | |

^a^ R^2^ de Nagelkerke.

^b^ Model (Forward LR).

^c^ Unstandardized coefficient.

^d^ Individuals who identified as black, mixed-race, indigenous, or other non-white categories.

^e^ who can read and write, demonstrating recognition of the alphabetic system.

^f^ Values were not calculated by the statistical program because the variable did not meet the criteria for regression analysis.

Younger: 60-70 years.

Older: >70 years.

Note: In this more detailed presentation of the binary logistic regression, we display each crude variable (Functionality, Depressive Symptoms, and Frailty) and adjusted for potential confounding factors (Skin Color and Education), along with their respective values from Stage 1 and the corresponding constants. For the adjustment analyses, the independent scalar variables Functionality (Lawton & Brody), Depressive Symptoms (GDS-15), and Frailty (EFS) were tested against the outcome variable (Risk of abuse and violence [H-S/EAST]), with each of them stratified according to the categories of the variables Skin color and Education within each study group. No significant variations in R² and OR were observed between the crude independent variables and their adjusted representations. This pattern was consistent across both study groups. Some values are not presented (-) as they were not eligible for the regression test. The summary of this analysis is presented in Table 3.
