# Supplementary material for: Sociodemographic profile, functionality, depression, and frailty as determinants for the risk of abuse and violence against older people in the community: An observational study conducted in Brazil
Source: PLoS One. 2025 Jun 16;20(6):e0317855. doi: 10.1371/journal.pone.0317855 (PMC12169517; doi:10.1371/journal.pone.0317855)
Supplement: S2 Table — (DOCX) [file pone.0317855.s002.docx]

**S2 Table. Collinearity coefficient analysis between the independent variables according to age group.**

| **Independent variables - Collinearity Coefficients** | | | | | | | | | | |
| --- | --- | --- | --- | --- | --- | --- | --- | --- | --- | --- |
| **Age group** | **Scalar Variables** | **Non-standard coefficients** | | **Standardized coefficients** | **t** | **p** | **CI 95% to ß** | **Collinearity Statistics** | |  |
|  |  | **ß** | **Standard Model** | **Beta** |  |  |  | **Tolerance** ^a^ | **VIF** ^b^ |  |
| Younger (n= 132) | Constant | 0.41 | 0.40 | - ^c^ | 1.006 | 0.316 | -0.39 – 1.20 | - | - |  |
|  | Functionality (Lawton & Brody) | -0.01 | 0.02 | -0.08 | -0.768 | 0.444 | -0.05 – 0.02 | 0.45 | 2.24 |  |
|  | Depressive Symptoms (GDS-15) | 0.10 | 0.02 | 0.63 | 6.343 | <0.001 | 0.07 – 0.13 | 0.50 | 1.99 |  |
|  | Frailty (EFS) | -0.02 | 0.02 | -0.12 | -1.017 | 0.311 | -0.06 – 0.02 | 0.33 | 3.00 |  |
| Older (n=68) | Constant | 1.14 | 0.42 |  | 2.717 | 0.008 | 0.30 – 1.98 | - | - |  |
|  | Functionality (Lawton & Brody) | -0.05 | 0.02 | -0.43 | -2.736 | 0.008 | -0.09 – -0.01 | 0.41 | 2.44 |  |
|  | Depressive Symptoms (GDS-15) | 0.06 | 0.02 | 0.36 | 2.439 | 0.018 | 0.01 – 0.11 | 0.47 | 2.12 |  |
|  | Frailty (EFS) - Scalar | -0.02 | 0.03 | -0.17 | -0.910 | 0.366 | -0.07 – 0.03 | 0.31 | 3.24 |  |
| Total (n=200) | Constant | 0.68 | 0.28 | - | 2.435 | 0.016 | 0.13 – 1.23 | - | - |  |
|  | Functionality (Lawton & Brody) | -0.03 | 0.01 | -0.19 | -2.183 | 0.030 | -0.05 – -0.03 | 0.43 | 2.33 |  |
|  | Depressive Symptoms (GDS-15) | 0.09 | 0.01 | 0.56 | 6.847 | <0.001 | 0.06 – 0.12 | 0.50 | 1.99 |  |
|  | Frailty (EFS) - Scalar | -0.02 | 0.02 | -0.16 | -1.547 | 0.124 | -0.05 – 0.01 | 0.32 | 3.17 |  |

^a^ Acceptable Tolerance: > 0.100
^b^ Variance Inflation Factor (VIF) Interpretation: VIF = 1.00 (no multicollinearity); VIF > 1.00 and < 5.00 (moderate multicollinearity); VIF > 5.00 and < 10.00 (high multicollinearity); VIF > 10.00 (Severe multicollinearity)

^c^ Not applicable.

Method: Enter

Younger: 60-70 years.

Older: >70 years.
