# Supplementary material for: Sociodemographic profile, functionality, depression, and frailty as determinants for the risk of abuse and violence against older people in the community: An observational study conducted in Brazil
Source: PLoS One. 2025 Jun 16;20(6):e0317855. doi: 10.1371/journal.pone.0317855 (PMC12169517; doi:10.1371/journal.pone.0317855)
Supplement: S3 Table — (DOCX) [file pone.0317855.s003.docx]

**S3 Table. Analysis of the interaction between the independent variable for each age group and total sample.**

| **Independents variables - Interaction history** ^a.b^ | | | | | | | |
| --- | --- | --- | --- | --- | --- | --- | --- |
| **Age Group** | **Stage** | **Interactions** | **2 Log Likelihood** | **Coefficients** | | | |
|  |  |  |  | **Constant** | **Functionality (Lawton & Brody) - Scalar** | **Depressive Symptoms (GDS-15) - Scalar** | **Frailty (EFS) - Scalar** |
| Younger (n= 132) | 3 | 1 | 129.424^c.d^ | -0.377 | -0.055 | 0.405 | -0.077 |
|  |  | 2 | 124.469 | 1.003 | -0.144 | 0.564 | -0.142 |
|  |  | 3 | 124.165 | 1.641 | -0.181 | 0.610 | -0.163 |
|  |  | 4 | 124.163 | 1.700 | -0.184 | 0.614 | -0.165 |
|  |  | 5 | 124.163 | 1.700 | -0.184 | 0.614 | -0.165 |
| Older (n=68) | 3 | 1 | 67.786^e.f^ | 2.570 | -0.201 | 0.241 | -0.092 |
|  |  | 2 | 66.659 | 3.374 | -0.258 | 0.311 | -0.123 |
|  |  | 3 | 66.638 | 3.527 | -0.268 | 0.322 | -0.129 |
|  |  | 4 | 66.638 | 3.531 | -0.268 | 0.322 | -0.129 |
|  |  | 5 | 66.638 | 3.531 | -0.268 | 0.322 | -0.129 |
| Total (n=200) | 3 | 1 | 201.802^g.h^ | 0.724 | -0.108 | 0.366 | -0.094 |
|  |  | 2 | 196.774 | 1.400 | -0.161 | 0.491 | -0.135 |
|  |  | 3 | 196.601 | 1.637 | -0.177 | 0.518 | -0.146 |
|  |  | 4 | 196.601 | 1.652 | -0.178 | 0.519 | -0.146 |
|  |  | 5 | 196.601 | 1.652 | -0.178 | 0.519 | -0.146 |

^a^Method: Enter

^b^Constant is included in the model.

^c^Initial -2 Log Likelihood: 126.094 for split file Age Group = Younger.

^d^Estimation terminated at iteration number 5 because parameter estimates changed by less than 0.001 for split file Age Group = Younger.

^e^Initial -2 Log Likelihood: 67.405 for split file Age Group = Older.

^f^Estimation terminated at iteration number 5 because parameter estimates changed by less than 0.001 for split file Age Group = Older.

^g^Initial -2 Log Likelihood: 199.272

^h^Estimation terminated at iteration number 5 because parameter estimates changed by less than 0.001.
