# Supplementary material for: Sociodemographic profile, functionality, depression, and frailty as determinants for the risk of abuse and violence against older people in the community: An observational study conducted in Brazil
Source: PLoS One. 2025 Jun 16;20(6):e0317855. doi: 10.1371/journal.pone.0317855 (PMC12169517; doi:10.1371/journal.pone.0317855)
Supplement: Attachment — Submitted filename: Response to Reviewers.docx [file pone.0317855.s005.docx]

**Response to Review**

Dear reviewers,

We sincerely thank you for all your thoughtful comments, which have greatly improved our work. We conducted an extensive and careful review and made every effort to address all the requests. Below, we provide point-by-point responses to each of the comments.

**Comments to the Author**

1. Is the manuscript technically sound, and do the data support the conclusions?

The manuscript must describe a technically sound piece of scientific research with data that supports the conclusions. Experiments must have been conducted rigorously, with appropriate controls, replication, and sample sizes. The conclusions must be drawn appropriately based on the data presented.

Reviewer #1: Yes

Reviewer #2: No

Reviewer #3: No

2. Has the statistical analysis been performed appropriately and rigorously?

Reviewer #1: Yes

Reviewer #2: No

Reviewer #3: No

3. Have the authors made all data underlying the findings in their manuscript fully available?

The [PLOS Data policy](http://track.editorialmanager.com/CL0/http:%2F%2Fwww.plosone.org%2Fstatic%2Fpolicies.action%23sharing/1/010f0193312a850e-8beed2da-00e6-4244-a173-217450b8196c-000000/4VmBBcfTotUnRav0wk19SfvEm3CP1Fl96PoljZ27ucs=185) requires authors to make all data underlying the findings described in their manuscript fully available without restriction, with rare exception (please refer to the Data Availability Statement in the manuscript PDF file). The data should be provided as part of the manuscript or its supporting information, or deposited to a public repository. For example, in addition to summary statistics, the data points behind means, medians and variance measures should be available. If there are restrictions on publicly sharing data—e.g. participant privacy or use of data from a third party—those must be specified.

Reviewer #1: Yes

Reviewer #2: No

Reviewer #3: No

4. Is the manuscript presented in an intelligible fashion and written in standard English?

PLOS ONE does not copyedit accepted manuscripts, so the language in submitted articles must be clear, correct, and unambiguous. Any typographical or grammatical errors should be corrected at revision, so please note any specific errors here.

Reviewer #1: Yes

Reviewer #2: Yes

Reviewer #3: Yes

5. Review Comments to the Author

Please use the space provided to explain your answers to the questions above. You may also include additional comments for the author, including concerns about dual publication, research ethics, or publication ethics. (Please upload your review as an attachment if it exceeds 20,000 characters)

**Reviewer #1:**

Dear Authors,
Generally the paper is interesting. The conceptualization, methodology, and description of the results are done well. The results are presented in the form of tables. The literature is up to date. The conclusions from the research are adequate to the results. My attention was drawn to the fact of the selection of the sample, there were more younger people than older than 70. After 70 years of age, the percentage of frail and depressed people is higher according to the research. The compared groups are too unequal. Please take these comments into account in the planned next studies.

**Answer:**

We thank the reviewer for the positive comments and for the observation regarding the sample composition. We recognize that the number of participants over 70 years old was smaller, which may limit comparisons between age groups. The disparity between groups occurred due to the use of the median for the cutoff analysis, as the variable "age" did not follow a normal distribution. The same criterion was applied to define nonparametric tests (association and binary logistic regression). However, this will be addressed in future studies to ensure a more balanced sample.

**Reviewer #2:**

1. The sample size calculation using "p" as 50% should give a sample size equivalent to 384. The sample of the study is 200. Please explain the sample calculation and the formula used.
**Answer:**
We appreciate the comment. After reviewing the calculation, we identified a typographical error in the previous version of the manuscript. The correct sample size is 384, not 200. Furthermore, due to data collection during the post-pandemic period, it was not possible to achieve the calculated sample size. This limitation has been included in the "Study Limitations" section of the manuscript, and we have corrected this information in the "Population and Sample" section of the Methods.

2. In instruments and variables section, please add the total score points for H-S/EAST and EFS frailty tools as well.

**Answer:**
As requested, we included the total scores for the scales.

3. The independent and dependent variables listed in line 182 and 183 seems to be interchanged in the manuscript. Please revise the variables accordingly.
**Answer:**

There was indeed an inversion between the independent and dependent variables. We made the necessary corrections to the text to ensure clarity and accuracy.

4. The study population have been categorized into two groups, namely, younger and older group. The sample size in the older group is too small that influences the statistical analysis and hence does not generate results that can be generalized.
**Answer:**

We acknowledge that the sample size for the older adult group was small, which may limit the robustness of statistical analyses and the generalizability of the results to this population. The disparity between groups occurred due to the use of the median for the cutoff analysis, as the variable "age" did not follow a normal distribution. The same criterion was applied to define nonparametric tests. This information has been detailed in the "Data Analysis and Processing" section of the manuscript to clarify the group disparity and has also been added to the study limitations.

5. The authors have not mentioned the sampling technique used to select the study participants. Please explain in detail which method was followed in the study.
**Answer:**

We used a non-probabilistic convenience sampling method to select the study participants. The PHC units provided a list of potential participants, including their addresses and phone numbers. Those who were contacted and agreed to participate scheduled appointments with the researchers based on their availability. This information has been added to the "Population and Sample" section of the manuscript to clarify the sampling method and increase the study's transparency.

6.Regarding the statistical analyses performed, please make clear if any confounders were adjusted in the logistic regression analysis and also explain why they were included. If not, I suggest to perform re-analysis adjusting for the variables so that the effect of the bias in the results can be controlled.
**Answer:**

We did not conduct additional analyses to adjust for confounding factors in the logistic regression analysis, as all aspects evaluated in the study are already reflected in the tables presented in the manuscript, directly representing the results obtained. Moreover, the variables included in the analysis were selected based on theoretical relevance and existing literature, ensuring that key factors were appropriately considered. For greater clarity and transparency, this information was added to the "Data Analysis and Processing" section of the manuscript.

**Minor comment:**

7. In line 288, the author may replace the term "thesis" into "study".

**Answer:**

We made the requested change.

**Reviewer #3:**

The paper is interesting and highlights the public health concern among the geriatric population and association with mental health (abuse and violence). However, the authors need to work more on several sections to improve the quality of the paper for the publication.

1. Abstract: The abstract section requires a minor revision in terms of methodology aspect.

**Answer:**

We added important details to clarify the methodological aspects of the study.

2. Introduction: The introduction section provides a global and local context burden of abuse and violence. However, it has not emphasized on the evidence on the burden among the geriatric population in both context.

**Answer:**
We added some Brazilian context data to the introduction.

Line 66-67 : Abuse and violence against the older people result from a multifactorial and
complex process, considered a severe public health problem. Can you elaborate more on these multifactorial and complex processes? Providing global data on abuse and violence among the similar study population could provide reader a clear picture of the burden of this issue.
**Answer:**
In addition to global data, we included the results of a conceptual analysis that examines the various aspects of this complexity.

3. Methodology: The methodology section needs to be expanded to provide details.

Ethical aspects : How was the written consent form obtained from the illiterate study participants? Did the data collection interfered their health service uptake hours?
**Answer:**

Written consent for illiterate participants was obtained by reading the consent form aloud to them, and, upon agreement, the participant's fingerprint was collected as a signature. No healthcare service hours were disrupted, as participants were approached at convenient times after their appointments, without interrupting the healthcare services provided. These details were clarified in the manuscript section.

Study design and location: More detailed information of the studied area and Brazil, that may be more valuable for readers to understand how representative this study is. Provide detail about the rational and sampling technique for choosing study site.
**Answer:**

The "Study Design and Location" section was revised to include more detailed information about the study area and Brazil. In the municipality, there are 29 health units distributed across five regions. A random lottery was conducted to select the data collection sites, and there was no refusal from the selected units. These details were added to provide a better understanding of the study's representativeness.

Population and sample: The sample size calculation need revision. Provide sufficient details about the sampling technique for the selecting the study participants. Provide details about how the sample size was approached in the form of write up or flow diagram. How many were approached initially? How many were eligible? How many did/did no provide consent?
**Answer:**

After the review, we identified a typographical error in the sample size calculation, which has been corrected in the text. The correct calculation indicated a sample size of 384 participants. A non-probabilistic convenience sampling method was used. Initially, 200 individuals were approached, all of whom were eligible and signed the consent forms. This process was carried out with the assistance of professionals from the primary health care units, who directed researchers to contact only those who had previously expressed interest through them. We had no control over the number of individuals who declined to participate. These details were added to clarify the sample size calculation process and the inclusion of participants in the study.

Data collection and availability: The paper does not clarify the data collection duration period. Please specify the data collection duration period. In which language was the data collection carried out? Was the language translated into the local language? Was the language understandable and comfortable to gather the needed information for the study?
**Answer:**

The data collection period occurred from April to July 2022. Data collection was conducted in Portuguese, the native language of Brazil, and no other language was required. These details were included in the "Data Collection and Availability" section to clarify both the data collection period and the language used.

Instrument and variables:

Line 146-147: To collect information, we used a sociodemographic and health data questionnaire, which was created and validated by the researchers themselves.

Sufficient details on how the questionnaire was validated is needed for clear understanding.
**Answer:**

The questionnaire was validated through pre-tests. Interviews were conducted with older adults who were not part of the study population. During these interviews, adjustments were made to the wording of the questionnaires before their actual application to the sample. These details were included to provide a clearer explanation of the questionnaire validation process.

Education variables as illiterate and literate needs to be explained for the readers ?
**Answer:**

Definitions of "illiterate" and "literate" were included according to the criteria used in Brazil in the relevant section. Additionally, this definition was added as a note in the table to ensure clarity for readers.

The authors have used the standard tools for abuse and violence, functionality, depression and frailty. But the manuscript lacks information regarding the validity and reliability of these tools. Any pretesting done for the reliability?
**Answer:**

We added the calculation of Cronbach's Alpha to measure the reliability of the scales applied together, which was considered substantial according to the theoretical framework we adopted. This information was added to the "Data Analysis and Processing" section.

Line 182-186: The risk of elder abuse and violence (H-S/EAST) was defined as the independent variable, and the dependent variables were gender, skin color, marital status, education, paid work, retirement, income, self-reported disease, number of medications, and the scores of functionality (Lawton & Brody), depression (GDS-15),
and frailty (EFS). Needs major revision in categorizing the dependent and independent variables.

**Answer:**

There was a typographical error, and the inversion of variables has been corrected in the text. The risk of elder abuse and violence (H-S/EAST) was defined as the dependent variable, and the other variables mentioned were defined as independent variables.

Data analysis and processing: Lacks details on data management and handling techniques. The use of two analytical test i.e chi-square and logistic regression to evaluate the association between independent and dependent variables is confusing for the readers. I suggest author to present the logistic regression analysis, mentioning the crude OR and adjusted OR. Was any variables adjusted for the confounding? If so, please mention the confounding variables that might influence the association.
**Answer:**

For the analysis of variables, nonparametric tests were used, starting with the chi-square test to explore possible associations, followed by binary logistic regression. Additionally, in Table 3, we included only variables that showed a relationship with the outcome, excluding those without significant results in the binary logistic regression analysis. To facilitate the interpretation of Table 3, the standard error (S.E.) of ß was added. Regarding the "confounding factor" variable, no variables were adjusted in the logistic regression analysis since all aspects evaluated in the study are already reflected in the tables presented in the manuscript, directly representing the results obtained. For greater clarity and transparency, this information was added to the "Data Analysis and Processing" section.

Results: This section can be improved by clearly stating the reference and comparison variables in the tables section.
**Answer:**

The suggestion has been addressed, and reference and comparison variables have been clearly highlighted in the table section.

Discussion: The paper would benefit from incorporating more detailed comparative and analytical perspective of the research findings rather than just mentioning the findings.

**Answer:**

The discussion section was revised to include a more detailed comparative analysis of the results, contextualizing them with previous studies.

6. PLOS authors have the option to publish the peer review history of their article ([what does this mean?](https://track.editorialmanager.com/CL0/https:%2F%2Fjournals.plos.org%2Fplosone%2Fs%2Feditorial-and-peer-review-process%23loc-peer-review-history/1/010f0193312a850e-8beed2da-00e6-4244-a173-217450b8196c-000000/FYJu9vnQqX64GNs2jeSjqpdX19R5RdYMYI7g2n6j91Q=185)). If published, this will include your full peer review and any attached files.

If you choose “no”, your identity will remain anonymous but your review may still be made public.

**Do you want your identity to be public for this peer review?** For information about this choice, including consent withdrawal, please see our [Privacy Policy](https://track.editorialmanager.com/CL0/https:%2F%2Fwww.plos.org%2Fprivacy-policy/1/010f0193312a850e-8beed2da-00e6-4244-a173-217450b8196c-000000/vTx_5WHIjlcQJI2PkvGUzFv0kpTw93Gg6q3akaQvcV0=185).

Reviewer #1: **Yes:** Marta Muszalik

Reviewer #2: **Yes:** Poonam Subedi

Reviewer #3: **Yes:** Surakshya Kunwar
