# Supplementary material for: Sociodemographic profile, functionality, depression, and frailty as determinants for the risk of abuse and violence against older people in the community: An observational study conducted in Brazil
Source: PLoS One. 2025 Jun 16;20(6):e0317855. doi: 10.1371/journal.pone.0317855 (PMC12169517; doi:10.1371/journal.pone.0317855)
Supplement: Attachment — Submitted filename: Response_to_Reviewers_auresp_2.docx [file pone.0317855.s006.docx]

**Response to Reviewers**

Dear Reviewers,

The responses to each of your concerns are detailed below. We hope to have fully addressed all of them, and we sincerely appreciate your feedback, as it has contributed to making our manuscript more robust.

-----------------------------------------------------------------------------------------------------------------

Reviewer #1: The authors made all corrections indicated by the reviewer. The research material and research results are worth publishing.
The conceptualization, methodology, and description of the results are done well.
The results are presented in the form of tables and figures.
The conclusions from the research are adequate to the results. The literature used is up to date
The authors highlighted this limitation at the end of their paper.

**Response:** We appreciate the reviewers' comments.
-----------------------------------------------------------------------------------------------------------------

Reviewer #2: Thank you authors for addressing the comments.
I still suggest to adjust variables to control any potential biases. The odds ratio if comes similar to binary logistic regression that you have used in this study, then it is okay.

**Response:** We have included a more detailed analysis considering the adjustments for potential confounding variables.
----------------------------------------------------------------------------------------------------------------

Reviewer #3: The Instruments and variables section needs attention in terms of presenting the variables in a more scientific way.

**Response:** We revised the text, and we believe it now presents a more appropriate language.

If the authors have not adjusted the confounding variables which might bias the results should be reported in the limitation of the study.

**Response:** We have added a more detailed analysis considering the adjustments for potential confounding variables
---------------------------------------------------------------------------------------------------------------

Reviewer #4: The manuscript could be improved based on the following comments:

Line 169: The information on questionnaire validation is to be provided.
The language version of all the questionnaires is to be highlighted, and the validation information is to be cited/presented - likewise the language used in the interviews.
**Response:** The bibliographic citations for each instrument in the text refer to the respective translation/validation studies for Brazilian Portuguese. To avoid repeating this information for each instrument, we have added a paragraph at the end of the "Instruments and Variables" section highlighting this point.

Line 138-141: The sampling method is cluster sampling. The sample size must consider the design effect (DE) to account for the increased variance due to clustering. The outcome variable where the sample size calculation was based on is to be mentioned.
**Response:** The sampling method was non-probabilistic and convenience-based, as described in the "Population and Sample" section. We have removed the statement that it was a cluster sampling, as we acknowledge this was an error.

Line 132-146: The flow description based on the sampling method and sample size calculation is unclear and requires revision.
**Response:** There was an inconsistency in the wording, which has been clarified in the previous response. With this correction, we believe the process is now described more clearly.

Line 141 & 145: The flow description between the sample sizes is unclear. Additionally, it is important to indicate whether the final sample size is adequate or underpowered following the application of the inclusion and exclusion criteria.
**Response:** The revision of the wording in response to the previous comments clarifies the sample flow description. Regarding the final sample size, we have added information to the manuscript stating that it represents a sample below the ideal representativeness.

Line 222-223: The statement ‘Quantitative variables were summarized with absolute (n) and relative (%) frequencies.’ is incorrect.
**Response:** We reviewed the sentence and believe it is now appropriately revised.

Line 225: The sentence requires revision.
**Response:** We have rewritten the entire paragraph to ensure a more technical and precise language.

Line 226-228: The sentence requires revision.
**Response:** We have rewritten the entire paragraph to ensure a more technical and precise language.

Line 229: The sentence requires revision. e.g the purpose of binary logistic regression is to be stated.
**Response:** The sentence was revised to align with the purpose of the test.

Line 231-232: The sentence requires revision. e.g. ‘For all tests, odds ratios (OR) were calculated, with an OR > 1.00 indicating a positive association between the exposure and the outcome.’
**Response:** The sentence was restructured according to the suggestion.

Line: 232-234: The sentence requires revision. e.g. A margin of error of 5.0% and a 95% Confidence Interval (CI) were adopted, with a significance level set at p<0.05 for all analyses.
CI 95% is to be written as 95% CI as the latter is more widely used and is a standard format in scientific writing.
**Response:** The sentence was restructured according to the suggestion.

Table 1: Number of Self-Reported Diseases and Number of Daily Medications: Typo error 0 a 2.

**Response:** Correction made.

There were no OR(95%CI) values presented for some variables.

**Response:** The analyses were reviewed, and the values have been added.

Health Characterization is to be written in a small cap.
**Response:** Correction made.

Line 231: Exp (B) and OR is to be standardized. E.g. Exp (B) is to be replaced with OR throughout.
**Response:** The requested substitutions have been made.

Line 235: What scales are this CA =0.695 is referring to?
**Response:** The raw value referred to the combined result of all scales. However, we have now added detailed values for each scale used in the study for better clarity.

A statement whether no missing or missing data (if any) is to be mentioned.
**Response:** There were no missing data in the responses for any of the scales. This information has been added to the manuscript.

The words crude and adjusted are to be utilized.
**Response:** The crude and adjusted values have been added.

Line 236-237: Not adjusting for confounders can lead to biased results, as other variables might distort the observed relationship. Usually, in best practice, logistic regression includes known confounders/significant confounders to control for their effects. Adjusted models will provide more reliable and valid estimates of the independent variable’s effect.
**Response:** We revised Table 3 and added six supplemental tables to provide a more in-depth analysis, considering adjustments for confounding factors. Additionally, we described the parameters used in the "Data Analysis and Processing" section under Methods. We believe these adjustments have added greater consistency and robustness to our results.

Line 283-289: The analysis approach needs to be reconsidered.
Tables 1 and 2 are to be stated as crude analyses, while Table 3 could be presented as an adjusted analysis.

**Response:** We have added the adjusted analysis in Table 3 and revised Tables 1 and 2.


Line 272, 274, 287, 289: The cosmetic presentation of figures is to be improved.
**Response:** We reorganized the information and adjusted the table formatting.

Line 293: Forward LR or Backward LR method is to be stated.
**Response:** We have included a mention of the Forward LR method.

Table 3: B is to be denoted in the table footnote, e.g., unstandardized coefficient. Wald to be included.

**Response:** Information added.

Thorough analysis assessing the collinearity and interaction could be done apart from assessing the confounding effects.

**Response:** The analyses have been referenced in the text and detailed in the Supplementary Information files.

In Table 1, education was found to be statistically significant in the crude analysis and could be considered for adjustment.

**Response:** The adjustment analyses have been made available in the Supplementary Information files.

It also needs to display the actual parameter estimate output that includes constant.

**Response:** The information has been presented as Supplementary Information.

The p for older group was not labelled with superscript ‘b’.

**Response:** Information added.


Tables 1, 2, and 3 presentation and formatting could be improved.

**Response:** We reorganized the information and adjusted the table formatting.


The list of references did not adhere to the journal's formatting guidelines.

**Response:** We reviewed the reference list and used a reference manager to adjust the formatting according to the journal's guidelines. Below, we provide the correctly formatted reference list with their respective numbering.

1.	World Health Organization. Un decade of healthy ageing: plan of action (2021-2030) Geneva, 2023 [Acess 2025 February 07]. Available from: https://cdn.who.int/media/docs/default-source/decade-of-healthy-ageing/decade-proposal-final-apr2020-en.pdf?sfvrsn=b4b75ebc_28.

2.	Brasil. Projeções da População do Brasil e Unidades da Federação: 2000-2070 Brasília-DF: Instituto Brasileiro de Geografia e Estatística; 2020 [Acess 2025 February 07]. Available from: https://www.ibge.gov.br/estatisticas/sociais/populacao/9109-projecao-da-populacao.html.

5.	Jandu JS, Mohanaselvan A, Johnson MJ, Fertel H. Elder Abuse. StatPearls: StatPearls Publishing; 2025.

7.	World Health Organization A, Life Course U, Université de Genève. Centre interfacultaire de gr. A global response to elder abuse and neglect : building primary health care capacity to deal with the problem world-wide : main report. Geneva: World Health Organization; 2008.

12.	Brasil. Disque 100 registra mais de 35 mil denúncias de violações de direitos humanos contra pessoas idosas em 2022 Brasília: Ministério dos Direitos Humanos e da Cidadania; 2022 [Acess 2024 June 3]. Available from: https://www.gov.br/mdh/pt-br/assuntos/noticias/2022/junho/disque-100-registra-mais-de-35-mil-denuncias-de-violacoes-de-direitos-humanos-contra-pessoas-idosas-em-2022.

13.	Brasil. Violências contra a pessoa idosa: saiba quais são as mais recorrentes e o que fazer nesses casos Brasília-DF: Ministério dos Direitos Humanos e da Cidadania; 2023 [Acess 2025 February 07]. Available from: https://www.gov.br/mdh/pt-br/assuntos/noticias/2023/junho/violencias-contra-a-pessoa-idosa-saiba-quais-sao-as-mais-recorrentes-e-o-que-fazer-nesses-casos.

37.	Fabricio-Wehbe SC, Schiaveto FV, Vendrusculo TR, Haas VJ, Dantas RA, Rodrigues RA. Cross-cultural adaptation and validity of the 'Edmonton Frail Scale - EFS' in a Brazilian elderly sample. Rev Lat Am Enfermagem. 2009;17(6):1043-9. doi: 10.1590/s0104-11692009000600018. PubMed PMID: 20126949.

40.	Landis JR, Koch GG. The measurement of observer agreement for categorical data. Biometrics. 1977;33(1):159-74. Epub 1977/03/01. PubMed PMID: 843571.

44.	Moura RF, Cesar CLG, Goldbaum M, Okamura MN, Antunes JLF. [Factors associated with inequalities in social conditions in the health of elderly white, brown and black people in the city of São Paulo, Brazil]. Cien Saude Colet. 2023;28(3):897-907. Epub 2023/03/09. doi: 10.1590/1413-81232023283.08582022. PubMed PMID: 36888872.

50.	Plante W, Tufford L, Shute T. Interventions with Survivors of Interpersonal Trauma: Addressing the Role of Shame. Clinical Social Work Journal. 2022;50(2):183-93. doi: 10.1007/s10615-021-00832-w.

53.	Brownell P. A reflection on gender in elder abuse research from a human rights perspective. Cien Saude Colet. 2016;21(11):3320. Epub 2016/11/10. doi: 10.1590/1413-812320152111.19972016. PubMed PMID: 27828564.

**Reference replaced with a more up-to-date version:**

30.	Brasil. Censo Demográfico 2022. Brasília-DF: Instituto Brasileiro de Geografia e Estatística.; 2023.

**Reference added in response to the requested changes.**

39.	DataCamp. Fator de inflação de variância (VIF): Como lidar com a multicolinearidade na análise de regressão New York: Data Camp; 2024 [2025 February 10]. Available from: https://www.datacamp.com/pt/tutorial/variance-inflation-factor.

- Ensure all the information ticked/checked in the STROBE statement checklist is included in the manuscript.

**Response:** We reviewed the STROBE statement and included it in the submission.
