# Supplementary material for: Sociodemographic profile, functionality, depression, and frailty as determinants for the risk of abuse and violence against older people in the community: An observational study conducted in Brazil
Source: PLoS One. 2025 Jun 16;20(6):e0317855. doi: 10.1371/journal.pone.0317855 (PMC12169517; doi:10.1371/journal.pone.0317855)
Supplement: Attachment — Submitted filename: Response_to_Reviewers_auresp_3.docx [file pone.0317855.s007.docx]

**Response to Reviewers**

Dear Reviewers,

We sincerely appreciate your new observations and suggestions. We have made every effort to fully address each one, and we believe that the manuscript is now even more robust and reproducible. Below, we provide detailed responses to each point raised.

-------------------------------------------------------------------------------------------------------------

Reviewer #2:

Dear Authors,
Thank you for addressing all the comments provided earlier.

You have conducted inferential statistics as recommended however no significant associations were found, which could also be because of smaller sample size.

**Response:** We appreciate the observation and fully agree with it. Therefore, we have added a statement acknowledging this potential influence in the study’s limitations section.

--------------------------------------------------------------------------------------------------------------

Reviewer #3: Thank you authors for addressing the comments.

The instrument and variables section is the most important part of the methodology section and reuqires more attention in terms of clarity. Please kindly present this section in a more scientific manner, highlighting the exposure and outcomes variables. A more detailed information regaring the pretested questionnaire, what kind of background researchers validated the questionnaire?

**Response:** We have clarified the classification of each variable as either an exposure or outcome variable, as requested. Regarding the pre-test, it was conducted by researchers with doctoral degrees in gerontology. This process has also been described in greater detail in the methodology section.

--------------------------------------------------------------------------------------------------------------

Reviewer #4: The analysis/statement in the following presentation needs to be revised.

S1 Table: The adjustment analysis is unclear. Explanation is to be provided in the table footnote.

**Response:** This table provides a more detailed breakdown corresponding to Table 3. We have included the constant values, as requested in the previous review. Additionally, we have added a note to the table with further clarification of the process and the results obtained, as suggested.

S2 Table, S3 Table: Usually collinearity analysis is done between independent variables.

**Response:** We reanalyzed the data, considering only the independent variables. While maintaining the division in the tables according to age groups, we have also added results for the total sample.

S4 Tab1e, S5 Table and S6 Table: Interaction is between independent t variables or interaction effect of independent variables on the dependent variable

**Response:** We have reanalyzed the data and now present a single table (S4 Table) showing the interaction among the three variables across the study groups and the total sample.

-----------------------------------------------------------------------------------------------------------------

We appreciate the reviewers’ valuable insights and remain available for any further clarifications.
